# The rise and flaws of the mission machine: A historical critique of research success metrics since the 1960s

**DOI:** 10.12688/openreseurope.20867.1

**Published:** 2025-08-28

**Authors:** Gabi Lombardo, Jonathan Deer, Poul Holm

**Affiliations:** 1 European Alliance of SSH, 54, Boulevard Raspail, 75006 Paris, France; 2 Research and Enterprise, City St. George's, University of London, London, EC1V 0HB, UK; 3 Trinity Centre for Environmental Humanities, School of Histories and Humanities, The University of Dublin Trinity College, Dublin 2, D02PN40, Ireland

**Keywords:** Frascati manual, science policy, OECD advice

## Abstract

Science policy systems depend on indicators to ensure that vast sums of money are spent in a transparent and accountable way. In this article, we investigate why and how the key accounting system, the Frascati manual, originated, was developed, and has been maintained over time, despite – and increasingly at variance with – developing policy objectives. The manual has undergone several updates and revisions but remains based on a set of parameters originally designed to measure research activity as it was performed and understood over 60 years ago. We argue that the manual is ill-equipped to capture fundamental changes in research resulting from breakthroughs in multidisciplinary approaches, digital technologies, and the co-creation of knowledge in partnerships in recent decades, and poorly equipped to measure the impact of the social and human sciences. In conclusion, science policy today is served by a systematically biased accounting system: in essence, a flawed Mission Machine.

## Plain language summary

Science policy relies on indicators to show how public money for research is spent. This article looks at the Frascati Manual—an international system used to track research funding. First created over 60 years ago, it has been updated several times but still reflects old ways of doing research. Today, research is often multidisciplinary, digital, and done through partnerships between universities, businesses, and communities. The manual struggles to account for these changes and does not measure the impact of the social sciences and humanities well. As a result, decisions about science funding are still based on an outdated system. The article argues that this creates a built-in bias in how we understand and support research. In short, today’s science policy is being guided by a tool that no longer fits the job—a flawed “Mission Machine” that needs urgent reform.

## Introduction

Research on the history of science funding policies often frames the emergence of science policy as part of postwar reconstruction efforts. This article, however, offers a new perspective by identifying the establishment of an accounting system—the Frascati Manual—as a key precondition that institutionalized rigid structures in research funding, making them more transparent and accountable but also resistant to change. Drawing on a historical analysis of national science policy systems in the 1950s and the influence of the UNESCO and OECD on funding objectives, we argue that the OECD’s implementation of a standardized accounting framework constrained national and European funding mechanisms in ways that remain difficult to alter. More significantly, it shifted decision-making authority over research priorities away from scientific communities and their overseeing ministries, placing it instead in the hands of finance ministries. While the approaches of these organizations differed slightly, the article highlights how the OECD’s rigid accounting system entrenched long-term structural effects in research funding governance.

## The development of global science policy systems

The roots of current science policy systems can be found in the World Wars of the first half of the twentieth century. The race to weaponise technology during World War I induced the military to establish strong scientific networks in France, Germany, Russia, and the U.S. Institutions such as CNRS in France and CNR in Italy originated in this environment, while strong links between the Russian Academy and the government were established during the war and carried on into Soviet times. A British attempt to align the academy with the government, however, failed, and German institutions were dissolved. ^1^

In the 1920s the League of Nations sought to develop science-based international treaties and multi-national academic collaboration. The International Institute for Intellectual Cooperation was set up to include professional scientific organizations and individual scientists active in international affairs. However, pleas in both the UK and the USA for increased budgets for research were not met by political support. It was only the Second World War that alerted politicians to the benefits of directing the best brains towards a common goal, the most spectacular result of which was the nuclear bomb. In July 1945, when the benefits of science were clear and the horrors still not in plain sight, the Director of the US Office of Scientific Research and Development, Dr Vannevar Bush, seized on this experience. In his document to the President, ‘Science – the Endless Frontier’, he proposed a peacetime National Research Foundation that would be dedicated to the combat of disease, to the security of the nation, and to job creation. Crucially, he devised a fuzzy rhetoric of ‘basic research’ that appealed both to politicians and scientists. While the former might understand ‘basic’ to meet national needs, scientists might understand the concept to indicate ‘pure’ or ‘curiosity-driven’ research. The tension between the two interpretations were embedded when the NSF was set up in 1950 and remains today. ^2^ The US model is still based on the pluralistic, multiple visions of the NSF and a series of mission-oriented agencies, primarily driven by security planning, and with priorities set by the defence programmes that have no civilian counterparts.

After World War II, science policy systems developed at pace around the world. By 1955 fourteen countries had such entities. It is often argued that the Cold War catalysed government interest in harnessing science for national objectives. ^3^ But as Robert Gilpin has pointed out this was not a straightforward equation. He argues that the French government developed a science policy in response to a perceived threat to French international influence and national independence from the USA after World War II. ^4^ In Japan, the end of the war marked a departure from the national objective of military strengthening. The country opted for economic-oriented aims and a science policy focused on industrial development. ^5^ The government's control over trade and foreign direct investment allowed a coordinated industry-government approach to obtaining technology from foreign companies in exchange for limited market access and licensing fees. The Japanese government strongly encouraged participation of the private sector in research and in the 1960s established a special funding program to support industrial consortia.

The French and Japanese examples illustrate that the security argument – so successful in US in 1950s - does not fully explain the fast uptake of science policy in many developed and developing countries. Strong international sentiments against the weaponization of science emerged in the aftermath of World War II, especially in Europe and in developing countries. In the late 1940s-early 1950s, UNESCO (United Nations Educational, Scientific and Cultural Organisation) encouraged its member states “to increase the world sum of scientific knowledge and access to that knowledge without regard to national boundaries”. ^6^ The aim was to avoid self-interested states from meddling with scientific priorities and academic freedom and prevent the exploitation of scientific discovery for military purposes. ^7^ This agenda was embraced by the International Council of Scientific Unions as well as private funders. An outstanding example was the international symposium on ‘Man’s Role in Changing the Face of the Earth’, organized in 1955 by the Wenner-Gren Foundation for Anthropological Research in Princeton, USA, which drew 52 talks representing a broad selection of geo- and biosciences as well as the social and human sciences. ^8^ Two years later the International Geophysical Year of 1957 epitomised the lofty goals of international collaboration. The collaboration of 67 countries, including the Soviet Union, seemed to indicate a thaw of the Cold War. Paradoxically, the launch of an artificial satellite, Sputnik, by the USSR in that year served to focus attention on security concerns and international conflict.

## The UNESCO and OECD models

Amidst this tension of research between security and academic freedom, UNESCO came to play a pivotal role by redefining science policy as “one of the foremost preoccupations of governments” as it was put in a 1960 report to the United Nations. Policy institutions were needed to ensure “interaction between the encouragement of scientific research, on the one hand, and economic and social progress, on the other”. ^9^ No argument was developed or presented at the time to require these policy institutions to demonstrate an improvement to the quality of science, an acceleration of scientific discoveries or indeed an increase in science capabilities. In short, science policy and research funding were to become an integral part of modern government, rather than being driven by bottom-up demand.

This position was not universally welcomed. The very first European conference of ministries of science took place in 1963 in Paris. The conference indicated that five countries (Belgium, the UK, Germany, France, and the Netherlands) had some form of national science policy. However, even these countries were sceptical about the idea of central authority setting priorities for science. As the Dutch representative maintained, “Allocation of funds should be left to scientists”. ^10^ The UNESCO approach was more welcomed in Latin America, Africa, the Middle East, and Southeast Asia. By 1975, eighty-nine countries had followed suit, establishing science policy and funding frameworks. ^11^ This rapid development was encouraged and closely monitored by UNESCO, which sent advisory missions and organised training seminars (see the case studies in Box 1). UNESCO’s advice covered institutional development as well as science policy issues.

UNESCO advocated for a strict separation between the formulation of science policy and the implementation of scientific research. This stance challenged existing early models, such as France’s CNRS and Italy’s CNR, by asserting that decisions on national research priorities should not be made by institutions that might prioritize their own interests. Instead, UNESCO argued that science policy should be set by a high-level governmental body—ideally within the Prime Minister’s or President’s office—to ensure broad authority and effective coordination. Placing it in a lower-ranking institution, such as a Ministry of Education, risked limiting its scope and influence.

While UNESCO firmly situated science policy within the nation-state framework—emphasizing that science should be organized for national benefit—this did not necessarily weaken the scientific community. On the contrary, many scientists gained greater access to government support and resources. However, a potential downside was that national priorities could sometimes conflict with the broader goals of international scientific collaboration.

**Box 1. UNESCO case studies**

**UNESCO: Lebanon a prototype of the model**

In the 1960s, UNESCO called for a large conference in Cairo to present their reports on the importance of having a coordinated research strategy at national levels. This prompted the Lebanese Foreign Affairs Ministry to request UNESCO's help in setting up a scientific research center, to be part of the University of Lebanon, which could carry out (rather than coordinate) scientific research in Lebanon in an efficient and effective way. The Director of the Natural Sciences Department at UNESCO maintained that Lebanon did not need another research centre but rather a proper coordination of their existing research efforts. He lobbied the President of Lebanon to establish a national research council that would organize research and make policy about science. However, the Lebanese government set up a national committee to work with a UNESCO science policy consultant to produce their own version of what UNESCO had already designed. In particular, they proposed that the new organism could still pursue independent research. The Director of Natural Science Department then removed the consultant and personally intervened to redraft a proposal for the Lebanese Parliament which was approved in 1962.

**East Africa**

Records from one of UNESCO's large subsequent science policy campaigns in East Africa during 1967 and 1968 provides some insight into the visits of consultants that UNESCO sent to Ethiopia, Tanzania, Zambia, Sudan to set up science administrations. Even in Zambia that did not require the UNESCO intervention directly, UNESCO officials intervened in the process. As a result, East African states had installed science policy administrations of a type in keeping with UNESCO's guidelines by 1970—within three years of the UNESCO consultants' initial visits.

Source: M. Finnemore, ‘International organizations as teachers of norms: the United Nations Educational, Scientific, and Cultural Organization and science policy’ International Organization 47, 4, Autumn 1993, 587

The rising costs of large-scale research placed financial considerations at the center of science policy debates. Traditionally, science policy had been seen as an extension of educational policy, falling under UNESCO’s domain. However, as research budgets grew, science funding became a concern for the international Organization for Economic Cooperation and Development. OECD economists began advocating for a clear separation between science funding and higher education, emphasizing the need for strategic national investment in research.

Alexander King, Secretary General for Scientific Affairs of OECD, commented in his autobiography that larger funds were needed as science was moving from supporting government needs and higher education to an investment for progress. ^12^ Furthermore, scientific development required larger and more strategic national investment. Costly infrastructures, national research labs, the increasing importance of discoveries for modern living and above all, the tension between fundamental and applied research were the roots for putting science investment as the basis of technological innovation and economic growth. 

The race for technological advancement drove several OECD countries to make two fundamental choices: divorce the investment in science from higher education and allow governments to set priorities for research funding at national levels. Some politicians, ministries and ambassadors continued to support the claim that science policy was inherently part of the educational policy and that it was therefore inappropriate for the OECD to drive national reviews. Some, in defence of better international collaboration, thought that science was still a matter for the UNESCO. ^13^

In 1963, the OECD published the influential Pigagniol Report, which laid the foundation for what became known as the ‘OECD model of science policy-making.’ ^14^. This and subsequent reports established standards for country reviews conducted by the OECD, focusing on key parameters such as horizontal coordination, planning and budgeting, priority-setting, resource allocation, and administration. A systems approach was introduced to streamline decision-making in science, technology, and innovation. ^15^

Between 1964 and 1974, several countries appointed science counsellors, reflecting the growing institutionalization of science policy. The 1971 OECD meeting in Paris further reinforced this shift, emphasizing the need for a "deliberate and coherent basis for national decisions" governing scientific investment, institutional structures, and research utilization. ^16^ Some nations went beyond creating science ministries—which assumed responsibility for funding priorities—and also established parliamentary scientific committees. The UK had pioneered this approach with its Parliamentary and Scientific Committee in 1939, but it was only in the early 1970s that such bodies began actively bridging national policy and science investment.

Yet, despite these institutional reforms, many countries struggled to align research efforts with broader societal goals. As Alexander King noted, science remained deeply entrenched in education, culturally ambiguous, and pervasive across government—making it difficult to integrate into coherent national strategies. "Efforts to shape constructive policies for science and its uses will long persist," he observed, "given the complexity of embedding science meaningfully into national priorities." ^17^

**Box 2. Case studies: Norway**

**Implementing OECD recommendations: Norway**

In 1967, Norway was one of the first countries to invite OECD officials to discuss how to design a research system, which address national interests. As Salomon recalls, the starting point was ‘with a false problem and a remarkable mobilizing slogan, we succeeded in diffusing the idea that science policy did not consist simply of pouring resources into fundamental research, but that it was necessary also to be preoccupied with the structure of the universities and with industrial innovation (Salomon, 3, 51).

In due course, four recommendations emerged. First, OECD believed that fundamental research should be directed towards ‘national goals’. Second, OECD insisted that research needed organization. Governments should not only set national goals, but also help the research system achieve them. Third, OECD put a strong emphasis on interdisciplinary research. What was needed was not narrow disciplinary knowledge deemed typical of the traditional universities, but knowledge drawn from different sources to solve practical-political problems. Fourth, OECD held that European academics, with their assumed rigidities, were failing to respond to national goals and social needs.

In the early 1960s, there were three research councils in Norway – the NAVF (Council for Science and the Humanities), responsible for fundamental research; the NTNF (Council for Science and Technology), which supported research for industry; and the NLVF (Council for Agricultural Research). In the 1970s, new councils were added for fisheries (NFFR) and for social planning (RFSP). Fundamental research was left to the universities, of which there were only two, Oslo and Bergen, until Trondheim and Troms, which came into being around 1970. In contrast to its Anglo-American counterparts, NAVF reflected a Continental model, and as such served the humanities as well as the natural sciences, the social sciences, and medicine. NAVF was virtually an extension of the universities, run by academics. In the early 1960s, it had a special obligation to safeguard fundamental research, and to remain independent of political direction.

However, following the OECD recommendations a new council was established: the Hovedkomiteen for norsk forskning (HK), (Central Committee for Norwegian Research). This Committee was at first welcomed as an ally, but its priorities – promoting research planning, applied research, and research for government and industry – were eventually to distance HK from NAVF. Some scientists were dismayed by the idea of planning, but the wider science community was torn between wanting to keep a safe distance from politics and wanting to use research to solve problems. In the late 1960s, as part of a deliberate policy of encouraging ‘sector research’, social scientists helped establish research as a permanent function of the Ministry of Social Affairs.

Source: E. Benum, Making Research Count: Norway and the OECD Connection 1965–1980. *Minerva* (2007) 45:365–387).

## Building a machine for science. An economic growth argument to invest in R&D

To effectively allocate funding and set priorities, national research systems needed measurable indicators. These metrics were essential not only to justify public spending on science but also to enable international comparisons. Before the 1950s, comprehensive measurement of Research and Development (R&D) was rare—most surveys focused either on industrial or government R&D in isolation, with little effort to combine them into a unified "national research budget." In 1939, the British scientist J. D. Bernal conducted one of the first systematic assessments of science expenditures in a Western nation. He calculated what he termed the "budget of science"—the total national spending on R&D—and proposed a groundbreaking metric: research expenditure as a percentage of national income. This measure later became a cornerstone of science and technology indicators.

The 1950s saw the rise of quantitative analysis in science studies, particularly through measuring growth and national accounting. Economists, including R. Solow in 1957, developed methods like econometric productivity models to quantify science and technology’s role in economic growth. ^18^ Other researchers measured the economic ‘cost’ of science by analysing its share of national income or budgets. ^19^

The case for standardized measurement gained momentum in 1959 when H. E. Stirner of Johns Hopkins University advocated to the NSF for a national R&D accounting system. He emphasized the critical links between R&D, technological innovation, and economic growth across sectors. ^20^ This perspective laid the conceptual foundation for the ‘National Innovation System’ framework that emerged in the 1980s, which viewed research as an integrated ecosystem of government, academia, and industry based on the idea that “the research system's ultimate goal is innovation”. ^21^

From the 1960s onward, as the OECD came to view science as a key driver of economic growth, the organisation emphasised that science policy must be grounded in reliable statistics as made clear in the Pigagniol report. Subsequent OECD country reviews revealed significant variations in how member states measured R&D expenditures and research personnel. These indicators were considered essential for three critical policy decisions, all expressed in the language of neoclassical economics:

- Determining the optimal allocation of resources to R&D
- Achieving balance among competing research priorities
- Assessing the efficiency and effectiveness of research activities

To standardize these measurements across nations, the OECD developed the Frascati Manual - a comprehensive methodological guide for national statisticians conducting R&D surveys.

## Accounting and science policy: the Frascati Manual

Since 1962, the Frascati Manual offers definitions, classifications and indicators for national statisticians for measuring the expenditures and human resources devoted to R&D. Also, it compiles comparable statistics among countries. According to the OECD, the manual “has probably been one of the most influential documents issued by this [Science and Technology] Directorate”. ^22^ The manual is now in its seventh edition (2015), and is the standard used in national statistical offices. “The objective of this manual is to attain international comparability in the narrower field of R&D (...). Arising from this experience, further international standards can be elaborated by the OECD for related activities”. ^23^

The first edition of the manual was based on a ‘moral’ hierarchy of scientific activities in vogue for decades: “The facilities available in the laboratories make it possible for the scientist to devote his time exclusively to *work of a professional caliber* [R&D]. He is not required to perform *routine* tasks of testing and experimentation but is provided with *clerical and laboratory assistants* who carry on this work”. ^24^ No argument was needed to convince people of this hierarchy. It was taken for granted by almost everybody that ‘soft’ activities like market studies or design, for example, were not part of science. In the second edition, the OECD turned to a precise definition of research: R&D is “creative work undertaken on a *systematic* basis to increase the stock of scientific and technical knowledge, and the use of this stock of knowledge to devise new applications”. ^25^

In other words, the Frascati manual developed three sets of guidelines. Firstly, norms were proposed for defining science as “systematic” research, ^26^ excluding some research activities (e.g. data collection, economic studies, market research etc.) or development for production, and research for teaching. Secondly, the manual suggested a classification of research activities based on the research funder or performer: government, university, industry or non-profit organizations, and the type or character of the research, either basic, applied or concerned with the development of products and processes, particularly when funded by industry or non-profit organisations. Thirdly, the research activities were classified by discipline in the case of universities (and non-profit organizations), by industrial sector or product in the case of firms, and by functions or socioeconomic objectives in the case of governments. ^27^

In 1993, the manual explicitly recommended concentrating on continuous R&D only: “R&D has two elements. R&D carried out in formal R&D departments and R&D of an informal nature carried out in units for which it is not the main activity. In theory, surveys should identify and measure all financial and personnel resources devoted to all R&D activities. It is recognised that in practice it may not be possible to survey all R&D activities and that it may be necessary to make a distinction between ‘significant’ R&D activities which are surveyed regularly and ‘marginal’ ones which are too small and/or dispersed to be included in R&D surveys. (...) This is mainly a problem in the business enterprise sector where it may be difficult or costly to break out all the ad hoc R&D of small companies.” ^28^

## Acceptance of the OECD Frascati Manual

The Frascati Manual not only standardized the definition and measurement of science but rapidly became the essential reference for policymakers and research institutions worldwide. Adopted as a foundational text by ministries, research councils, and funding agencies, it shaped how nations account for research investment. Science policy experts formed specialized working groups to refine its technical implementation, establishing consensus around global best practices. Over half a century later, the Manual remains the authoritative guide for Science and Technology (S&T) professionals and government officials in quantifying and managing national research expenditures.

Three key factors facilitated the widespread adoption of the Frascati Manual among OECD countries. First, in the early 1960s, most nations lacked systems for collecting science data. The Manual provided an immediate solution for these countries, while also aligning with existing practices in the few that did collect such data (notably the United States, Canada, and the United Kingdom). This created a shared framework that accommodated both newcomers and experienced practitioners. Second, the Manual’s development under an international organization rather than a single country lent it an air of neutrality—despite significant U.S. influence. This multinational endorsement enhanced its credibility and acceptance across member states. Third, the OECD implemented a carefully phased introduction strategy:

- Initial development (1962): The first edition circulated internally as an unofficial document
- Field testing (1963–64): Extensive trials in multiple countries
- Refinement and institutionalization (post-1964): Regular revisions based on survey results, with official publication delayed until the third edition (1976)

An additional crucial factor was the active participation of national statisticians in shaping OECD standards through:

- The National Experts on Science and Technology Indicators (NESTI) group, which guided OECD activities
- Periodic ad hoc review groups that ensured statistics met user needs
- Direct member country collaboration in developing specific indicators.

This collaborative, evolutionary approach secured enduring international agreement on the Manual's framework. For six decades, the statistical representation of science developed through this process has profoundly shaped policymaking. By providing standardized accounting tools, it established a dominant paradigm for science policy - one that prioritizes research management through quantifiable economic outcomes. Governmental and international organizations have consequently come to view and evaluate science primarily through measurable returns on investment. ^29^

The OECD's country reviews initially promoted GDP-based comparisons of science investment, but this approach faced limitations. Many nations found GDP metrics problematic due to varying national priorities and policy differences, preferring instead to measure R&D personnel per capita. This led the OECD to expand its data collection in the early 1970s, aiming to incorporate socioeconomic objectives of government-funded R&D into the third Frascati Manual (1975). The methodology for this expansion came from an initiative by the European Commission to collect data to design a European science policy and budget. In 1968, the European Commission's Working Group on Scientific and Technical Research Policy established a statisticians' group to analyse government R&D funding motivations. ^30^ Their work produced the Nomenclature for Analysis and Comparison of Science Programmes and Budgets (NABS) in 1969, creating Europe's first standardized framework for science policy design. ^31^

While the OECD's Directorate for Science, Technology and Industry (DSTI) adopted this European approach, ^32^ implementation proved challenging. Most governments resisted conducting specialized R&D surveys, ^33^ opting instead to use existing budget documents despite their lower accuracy, primarily due to cost and convenience considerations. ^34^

OECD statisticians faced significant challenges in harmonizing diverse national budgeting systems across member states. The classification units varied substantially between countries due to fundamental differences in budgetary procedures and documentation. Germany maintained particularly detailed budget records that enabled precise attribution of research objectives, while the United Kingdom and Canada relied on surveys of funding agencies that already used international classifications. France's system presented different complications, with research funding primarily organized by ministerial votes rather than functional categories. These structural variations limited the OECD's ability to impose uniform standards across national accounting practices. ^35^

A proposal that nations allocate 20% of R&D funding to basic research became an influential benchmark in OECD policy discussions. ^36^ This target relied on classifying expenditures into basic research, applied research, and development - a distinction enabled by the development of Gross Domestic Expenditure on R&D (GERD) metrics, with GERD/GDP ratios emerging as key policy indicators.

For four decades (1962–2002), the Frascati Manual maintained a distinction between basic research (knowledge-focused) and applied research (application-focused). However, these terms faced persistent criticism and occasional rejection in practice. During the manual's fifth revision, member states expressed strong interest in refining the basic research category to distinguish between pure and oriented basic research, ^37^ but deferred substantive changes pending further policy analysis. ^38^ A workshop in 2001 on basic research funding did not substantially progress the issue. ^39^ In 2003 the OECD concluded that “the key question is not to find a new conceptual definition for basic research, but to define its scope sufficiently broadly to cover the whole range of research types needed to establish a sound body of knowledge to achieve socio-economic advances.” ^40^

The practical consequence of the lack of clarity was that governments systematically omitted basic research questions from surveys starting in the mid-1970s. This trend has persisted, with only about half of OECD members currently maintaining basic research data collection. Today, OECD publishes only aggregate sector-level statistics on basic research, primarily due to two persistent challenges: inconsistent data quality across nations and widespread non-compliance with data collection protocols among member states.

From its inception, the Frascati Manual emphasized the need for comprehensive metrics encompassing both input and output indicators to effectively measure scientific activity. Initial editions proposed output measures such as patents, licensing fees, and technical know-how payments. ^41^ By 1981, the manual expanded this framework significantly, dedicating an entire appendix to output measurement that incorporated additional indicators: technological innovations, patent activity, royalty payments, high-tech trade data, and productivity metrics. This evolution reflected a notable shift in tone. While acknowledging persistent measurement challenges, the manual adopted a pragmatic stance, arguing that imperfect data should not disqualify potentially useful indicators: "The difficulties inherent in using such data should not preclude their application, as they currently represent the only available means of quantifying research outputs." ^42^

The 1981 edition of the Frascati Manual reframed its introduction to situate R&D statistics within the broader landscape of science and technology indicators. This revision incorporated UNESCO's newly adopted concept of Science and Technological Activities (STA), defined as systematic efforts to generate, advance, disseminate, and apply scientific knowledge across all fields. While STA encompassed R&D, scientific training (STET), and technological services (STS), ^43^ the OECD maintained its exclusive focus on measuring R&D, explicitly distinguishing it from other STA components. ^44^ This conceptual expansion had limited impact on core measurement practices but introduced a transformative element: innovation. Unlike other STA categories, innovation gained unique standing in OECD statistics, achieving parity with R&D. This recognition culminated in the 1997 Oslo Manual (developed with Eurostat), the first dedicated framework for innovation measurement, following five years of drafting since 1992. ^45^

The Frascati Manual initially focused exclusively on natural and engineering sciences. Even the inclusion of higher education in GERD calculations required explicit justification, noting universities' dual role as hubs for fundamental research and crucial partners in national R&D policy development. ^46^

The 1976 edition marked a significant expansion by incorporating social sciences and humanities, though it acknowledged these fields often lacked the systematic research approach characteristic of STEM disciplines. ^47^ This inclusion necessitated a broader R&D definition: “ *R&D may be defined as creative work undertaken on a systematic basis to increase the stock of scientific and technical knowledge, including knowledge of man, culture and society and the use of this stock of knowledge to devise new applications*”. ^48^ Despite this formal recognition, implementation challenges persisted. A dedicated annex addressed measurement difficulties arising from the less structured nature of humanities research. The manual ultimately accepted these disciplines with caveats, explicitly stating that “some deviations from the standards may still have to be accepted”. ^49^

## Knowledge-based society and measuring innovation: the Oslo Manual

In the late 1990s, scholars and policy makers supported an emerging approach focused on the concept of the ‘knowledge-based society’ at the heart of which was innovation. The challenge was how innovation could be assessed. David and Foray argued: "A system of innovation cannot be assessed only by comparing some absolute input measures such as research and development (R&D) expenditures, with output indicators, such as patents or high-tech products. Instead, innovation systems must be assessed by reference to some measures of the use of that knowledge……The development of new quantitative and qualitative indicators (or the creative use of existing ones) is an urgent need in the formation of more effective science and technology policies". ^50^ The criticism was to some extent acknowledged as valid and the OECD National Innovation System project ^51^ considered indicators on knowledge distribution. However, it concluded, "it has proved difficult to produce general indicators of the knowledge distribution power of a national innovation system". ^52^

The Frascati Manual’s accounting framework faced particular challenges in capturing innovation and industrial research. Industrial R&D measurement prioritized systematized research conducted by large organizations with dedicated laboratories, ^53^ as identified through industry surveys. Given the impracticality of surveying all firms, the methodology introduced a significant sampling bias: while it comprehensively covered major R&D performers (large firms with formal research units), it only selectively included smaller enterprises, if at all. This approach received justification from the observable fact that large corporations typically maintained precise R&D accounting through distinct laboratory entities, unlike smaller firms. Furthermore, the surveys explicitly excluded activities like economic studies and market research from qualifying as R&D. ^54^ Consequently, this measurement method systematically underrepresented research activities that (1) occurred outside formal laboratory settings, and (2) characterised disciplines like humanities and social sciences that often rely on individual scholars or small research groups rather than large-scale organizational structures.

In 1991 in Oslo, the OECD Working Party of National Experts on Science and Technology Indicators (NESTI) sealed a first agreement within the global community of practitioners on how to conceptualise and measure business innovation. This agreement led to the publication of the Oslo Manual, developed with European Union support, which established guidelines for innovation surveys. The manual's implementation, facilitated by OECD and EU initiatives, enabled over 80 countries to systematically track innovation activities, including those of small and medium enterprises and other smaller-scale innovators. In later years, the OECD and Eurostat jointly led further revisions to the manual, extending its scope and increasing its methodological rigour. The fourth edition of the *Oslo Manual* (2018) takes account of major trends such as the pervasive role of global value chains, the emergence of new information technologies and their influence on new business models; the growing importance of knowledge-based capital, and the progress made in understanding innovation processes and their economic impact.

## Discussion: the limitations of the Frascati Manual in capturing contemporary research practices

Since its introduction in 1963, the Frascati Manual has functioned as the global standard for measuring research and experimental development (R&D). However, its traditional framework—designed for industrialized economies—struggles to capture contemporary innovation practices, resulting in the systemic underrepresentation of diverse forms of knowledge production in science and technology policy.

While complementary frameworks like the Oslo Manual (for innovation) and the OECD’s Going Digital project have sought to address these gaps, fundamental tensions remain. Horizon Europe, the Research Exercise Framework in UK, and the Canadian Research and Development Classification have made initial attempts to go beyond some standard practices but often changes of the overall approach to research funding and expanded the scale of project sizes were influenced not so much for better and more relevant science output, but rather for cost saving strategies. Leading institutions including the European Commission and OECD, along with national governments, now recognise fundamental flaws in existing assessment systems. ^55^ The issues are threefold:

- The GERD/GDP ratio and Frascati framework often distort rather than reveal true research investment trends
- Current measurements fail to capture critical research activities, undermining their usefulness for identifying scientific priorities
- The traditional economic growth rationale for research funding is showing significant limitations.

The manual prioritises institutionalized, monetized R&D, overlooking decentralized and participatory innovation such as crowdsourcing, open-source collaboration, and citizen science, which lack formal structures but contribute significantly to fields like public health and astronomy. Similarly, informal R&D by SMEs without dedicated infrastructure is often excluded from national statistics.

Contemporary digital and data-driven research challenges the manual’s classifications. Practices such as exploratory data mining, hackathons, prototyping, and agile development do not conform to the manual’s criteria of “systematic” work. User-driven innovation, including co-creation with end-users in healthcare or agriculture, remains inadequately recognized.

Moreover, the manual’s STEM-centric orientation marginalises non-technological research. Design thinking, behavioural science, and arts-based inquiry are often dismissed as ‘non-systematic’, despite their societal relevance. Cross-sectoral work in NGOs, CSR programs, and grassroots organizations is similarly neglected. Civic knowledge production—such as citizen science and traditional ecological knowledge—lacks institutional visibility, perpetuating colonial epistemic biases.

Finally, the manual inadequately addresses mission-oriented and transdisciplinary research aimed at global challenges. SDG-aligned initiatives often integrate scientific, policy, and community domains, blurring distinctions between R&D and implementation. Evidence generation by think tanks and advocacy groups, though vital for policymaking, is rarely classified as R&D. Today, the value of research for economic growth has become secondary to the need for collaboration to tackle common societal challenges. The Sustainable Development Goals extends this imperative to wealthy industrialised countries and calls upon science and researchers to achieve a society that prioritises wellbeing over financial returns. However, this is still highly disputed, especially with the need to fund a European Defence Agency and pressure to address urgent energy crises.

Global challenges relating to the consumption of ‘common pool’ or ‘common property’ resources require shared innovation efforts across the planet to be effectively addressed. Currently, however, there is no framework for organising such multilateral collaboration. In multiple studies, Elinor Ostrom demonstrated that common pool resources can be managed collectively through negotiation and collaboration among 'polycentric' agents. ^56^ The current notion implicit in science policy that science is a national resource to be developed by individual states differs greatly from UNESCO's original idea of science as a transnational enterprise, as well as from Ostrom’s recommendations.

Today, science policy mechanisms lack two fundamental requirements: instruments to capture and fund the breadth of knowledge that society needs, and an ‘evolution of institutions for collective action’. ^57^ This bias also continues in other OECD methodological manuals, ^58^ where a system of ‘priorities’ is established with the natural sciences and engineering situated at its core. ^59^ Although official definitions and surveys have begun to cover the social sciences and humanities, ^60^ the conventions designed for the natural sciences in the previous decades are still strictly applied. Consequently, activities such as data collection and the production of scientific and technical information, including the production of statistics and longitudinal studies, which form the basis of research in the social sciences and humanities, continue to be excluded because they are considered as ‘related scientific activities’. ^61^

Currently, there are no alternatives to the Frascati Manual. The Oslo Manual still aims at national accounting of innovation with more soft criteria linked to organisation skills and knowledge production for example, but it is still focused on investing in research as a function of industrial development for national interests. Emerging databases on universities and research personnel have provided some important data. For example, the ETER (Establishing Tertiary Education Registry) database is providing a new range of data in research personnel. Although this database is focused on the design of education policies, it provides new insights into universities contributions, beyond the limitations of large laboratories.

Education, science, and culture should draw a divided world of nation-states together. However, developing a new system of research accounting to underpin knowledge production would be a significant challenge. Not only would changes to the current system be costly, they would also produce new data that would not be comparable with previous years, thus altering one of the main purposes of the Frascati Manual: to collect comparable standards for R&D. Thus, despite the potential benefits of a new Mission Machine, there will be strong resistance to radically changing the current systems for research accounting and assessment at national levels. Such resistance to bureaucratic change will result in systematic bias in the information used for science policy.

Changing a system which has been developed and embedded for over 60 years is not an easy task. First, it requires the identification of a valid alternative. Currently countries remain relatively locked within their national accounting and reluctant to consider deep change not just at ministerial level, but also from within institutions like universities and research centres that provide the information. Second, any new system must be viable. Current options under examination by several countries are considered still too expensive. Third, a whole generation of policy officers and officials will need training in new indicators and how to use them. As experience has demonstrated, when a system identifies that the tools available do not deliver the desired outcomes, it does not necessarily mean that the system is able to change.

## Ethics and consent

Ethical approval and consent were not required.

## Data Availability

No datasets required for this article type.
