# Supplementary material for: The rise and flaws of the mission machine: A historical critique of research success metrics since the 1960s
Source: Open Res Eur. 2026 Aug 4;5:262. Originally published 2025 Aug 28. [Version 2] doi: 10.12688/openreseurope.20867.2 (PMC13598400; doi:10.12688/openreseurope.20867.2)
Supplement: Peer review history

# Reviewer report: Reviewer response for version 2

**DOI:** 10.21956/openreseurope.26673.r79425

**Published:** 2026-10-08

**Authors:** Carter Bloch

**Affiliations:** 1 Aarhus University, Aarhus, Denmark

This paper examines the Frascati Manual from a historical perspective, offering a critique of its role in science policy. It provides an interesting account of how R&D measurement, along with broader measurement of knowledge creation, has evolved over time, and points out limitations to measurement frameworks in their ability to capture different types of research practices.

 As the paper notes, what is or is not measured in statistical frameworks can matter for science policy making. However, the paper appears in many cases to go on to view the Frascati Manual as a research evaluation tool, which I would disagree with. It is a measurement framework, not evaluation framework that measures research performance or impact.

 It is argued that the measurement framework in the Frascati Manual was built to fit into existing accounting practices, though it is not clear what the alternative would be. While it is of course true that the Manual offers a framework to measures quantitative expenditures on R&D, the approach did not build on any existing measures that companies or universities already had in the financial accounts. 

 Arguments that current measurement of R&D is focused on systematic R&D activities and thus is not equipped to capture ad hoc, informal or non-systematic R&D seems well-founded, though is not based on any evidence or sources. For other cases, better measurement of aspects such as collaborative research, the international dimension of research, inter- and transdisciplinary research, and the pursuit of societal goals, no alternative is suggested. Arguably, these aspects can be covered by building on current frameworks, of which there are a number of examples.

Is the argument persuasive and supported by appropriate evidence?

Partly

Is the work clearly and cogently presented?

Partly

Is the topic of the essay discussed accurately in the context of the current literature?

Partly

Does the essay contribute to the cultural, historical, social understanding of the field?

Yes

Reviewer Expertise:

Research and innovation policy analysis; research and innovation measurement

I confirm that I have read this submission and believe that I have an appropriate level of expertise to confirm that it is of an acceptable scientific standard, however I have significant reservations, as outlined above.

# Reviewer report: Reviewer response for version 2

**DOI:** 10.21956/openreseurope.26673.r78616

**Published:** 2026-09-22

**Authors:** Merle Jacob

**Affiliations:** 1 Universitetet i Bergen Senter for vitenskapsteori (Ringgold ID: 213771), Bergen, Hordaland, Norway

**“The rise and flaws of the mission machine: A historical critique of research success metrics since the 1960s**” makes the historical-policy argument that the Frascati Manual became deeply embedded in research governance because it supplied governments with standardised, internationally comparable R&D statistics, but the assumptions built into that system increasingly misrepresent contemporary research. In particular, the authors argue that it privileges formal, institutionalized and economically legible R&D while inadequately capturing multidisciplinary, digital, participatory, social-science and humanities research.

 The paper contains a number of interesting historical insights but misses the point on a number of issues. Overall, the attempt to extrapolate from history to contemporary policy is the piece's greatest weakness. 

 The discussion argues that citizen science, open-source collaboration, traditional ecological knowledge, arts-based inquiry and other forms of knowledge production are inadequately captured.  This is a plausible assertion but there is no evidence to suggest that it is correct. In order to evidence this, one would have to show that the inclusion of these categories would make a difference.

 The critique offers no alternatives. 

 Frascati is a measurement framework, it is not a research evaluation system. The argument does not always keep these two entities apart. 

Is the argument persuasive and supported by appropriate evidence?

Partly

Is the work clearly and cogently presented?

Yes

Is the topic of the essay discussed accurately in the context of the current literature?

Partly

Does the essay contribute to the cultural, historical, social understanding of the field?

Partly

Reviewer Expertise:

Resarch policy and research funding

I confirm that I have read this submission and believe that I have an appropriate level of expertise to confirm that it is of an acceptable scientific standard, however I have significant reservations, as outlined above.

# Reviewer report: Reviewer response for version 2

**DOI:** 10.21956/openreseurope.26673.r78564

**Published:** 2026-08-05

**Authors:** Mar Rubio-Varas

**Affiliations:** 1 Public University of Navarra, Pamplona, Spain

No further comments

Is the argument persuasive and supported by appropriate evidence?

Partly

Is the work clearly and cogently presented?

Yes

Is the topic of the essay discussed accurately in the context of the current literature?

Yes

Does the essay contribute to the cultural, historical, social understanding of the field?

Yes

Reviewer Expertise:

Economic History, Socio-technical systems

I confirm that I have read this submission and believe that I have an appropriate level of expertise to confirm that it is of an acceptable scientific standard.

# Reviewer report: Reviewer response for version 1

**DOI:** 10.21956/openreseurope.22576.r59522

**Published:** 2025-09-16

**Authors:** Mario Coccia

**Affiliations:** 1 CNR-National Research Council of Italy, IRCRES-CNR, Turin Research Area of the CNR, Arizona State University, Turin, Italy

The rise and flaws of the mission machine: A historical critique of research success metrics since the 1960s

 The topics of this paper are interesting, to discuss about some limits of the Frascati Manual in contemporary science and research policy. 

 To re-design a new manual with best practices is not easy, as observed. It should consider the balance between public and private research, crowding out effects, as it has happening in space industry  with competitive driving forces, technological innovation, and unprecedented market opportunities. New emerging markets are developing also with the invasive wave of generative artificial intelligence. A new approach should be more and more oriented to sustainability. 

 The title has to be shorter and clearer. 

 Abstract has to clarify the goal, methods and implications for next science and research policy. 

 Introduction has to better clarify the research questions of this study, indicating the gap presents in literature that this study endeavors to cover, and provide more theoretical background. After that authors can focus on the topics of this study to provide a correct analysis for fruitful discussion. (See suggested readings that must be all read and used in the text). 

 Some comments to consider are the following. 

 A renewed science and research policy must reflect the profound transformations underway in both the technological and institutional landscapes. The traditional frameworks—such as the Frascati and Oslo Manuals—are increasingly misaligned with the realities of contemporary innovation ecosystems. These systems were designed to measure R&D in industrialized economies during the mid-20th century, focusing on formal, monetized, and institutionalized activities. Today, however, knowledge production is far more diverse, decentralized, and transdisciplinary.

 Modern science policy must embrace this complexity. It should recognize informal and participatory innovation practices—such as citizen science, open-source development, hackathons, and co-creation with end-users—as legitimate and impactful. These activities, though often lacking formal structures, contribute significantly to fields like public health, environmental monitoring, and digital innovation. Moreover, the policy must integrate mission-oriented research that addresses global challenges, such as climate change, health crises, and social inequality. These initiatives often blur the boundaries between research, implementation, and policy-making, requiring new models of evaluation and funding.

 The emergence of the New Space economy exemplifies the need for such a shift. Space is no longer the exclusive domain of government-led geopolitical activities; it is rapidly evolving into a competitive arena for private companies, SMEs, and start-ups. Technological breakthroughs—such as reusable rockets, satellite-based Earth observation, and space-enabled services—are transforming industries and creating new markets. The decreasing cost of space launches is democratizing access to space, enabling applications in energy, agriculture, logistics, and even tourism. These developments are accompanied by institutional shifts, changing market dynamics, and evolving regulatory frameworks.

 Science policy must therefore be capable of capturing the dynamics of these emerging sectors. It should support the development of space-enabled technologies, innovative services, and new business models. This includes fostering entrepreneurship, facilitating public-private partnerships, and enabling international collaboration. Agencies like NASA, ESA, and the European Commission are already playing a role in commercializing space through contracts, subsidies, and incubation programs. However, policy must also address the risks of monopolistic behavior and ensure that SMEs and start-ups can participate meaningfully in the space economy.

 Generative AI (GenAI) represents another frontier of transformation. Rapid advances in GenAI are revolutionizing operational processes across industries—from healthcare and finance to manufacturing and entertainment. GenAI enables the automation of content creation, the generation of complex data, and the enhancement of machine learning workflows. It simplifies product design, improves quality management, and accelerates innovation. Science policy must recognize GenAI as a critical enabler of future research and industrial competitiveness. It should support the integration of GenAI into scientific workflows, promote ethical and inclusive AI development, and ensure that regulatory frameworks keep pace with technological change.

 In this context, traditional metrics such as GERD/GDP ratios and publication counts are insufficient. They fail to capture the societal relevance, environmental sustainability, and collaborative nature of modern research. New indicators must be developed to assess impact, inclusivity, and mission alignment. These metrics should be capable of evaluating both quantitative and qualitative dimensions of research, including long-term and non-linear outcomes.

 Governance models must also evolve. Inspired by Elinor Ostrom’s principles for managing common-pool resources, science policy should promote polycentric and collaborative structures. It should facilitate multilateral cooperation, shared missions, and pooled resources. UNESCO’s vision of science as a global public good must be revitalized, emphasizing the role of science in fostering international solidarity and addressing shared challenges.

 The marginalization of the social sciences and humanities remains a critical issue. Despite their essential role in understanding societal dynamics and informing policy, these disciplines are often underfunded and undervalued. A renewed policy must ensure their full integration into research agendas, funding schemes, and evaluation systems. This includes recognizing the scientific value of data collection, longitudinal studies, and qualitative research methods.

 Developing a new accounting framework for research is a complex but necessary endeavor. While the current system offers comparability across time and countries, it also perpetuates systematic bias and exclusion. A new framework should be modular and flexible, allowing for contextual adaptation while maintaining continuity with historical data. Emerging data sources, such as the ETER database, offer promising avenues for capturing a broader spectrum of research activity, particularly in higher education.

 Ultimately, a future-oriented science and research policy must be inclusive, adaptive, and mission-driven. It should reflect the complexity of contemporary knowledge production, support emerging technologies and industries, and guide collective action toward a more just, sustainable, and collaborative future. This policy must be capable of navigating the intersections of technological innovation, institutional change, and societal need—ensuring that science serves not only economic growth but also human wellbeing and planetary health.

 Describe lessons learned, proposing recommendations that can help improve new science policy, highlighting best practices. In particular, science must be recognized as a global public good, supporting societal wellbeing and sustainability over narrow economic returns. The policy should integrate emerging sectors such as the New Space economy and generative AI, fostering innovation through public-private partnerships, SME engagement, and international cooperation. It must also redefine R&D to include informal, decentralized, and user-driven practices, ensuring visibility for citizen science, arts-based inquiry, and social sciences.

 New metrics are needed to assess impact, equity, and relevance, moving beyond GDP ratios and publication counts. Governance should be polycentric, enabling collective action across borders and sectors. A modular accounting framework must be developed to reflect diverse research activities while maintaining historical comparability.

 This policy envisions a science ecosystem that is agile, inclusive, and aligned with global missions—capable of addressing the urgent needs of our time through collaborative, ethical, and forward-looking research.

 Conclusion has not to be a summary. The Conclusion does not adequately discuss the theoretical and policy implications. Discuss how the gap in the literature has been addressed about the research problem.

 Create  3 subsections in the Conclusion: 1) Theoretical Implications, 2) Policy Implications, and 3) Ideas for Future Research.

 Overall, then, the paper is interesting, but the  paper has to be improved and clarified using suggested comments.

 I strongly suggest improving the paper by using all comments. 

 Suggested readings of relevant papers that have to be read, and all inserted in the text and references.

 Heikkilä, and W. D. Heavenarchive, “What’s next for AI in 2024,” MIT Technology Review, Jan., 2024. Accessed: Sep.17, 2024.

 Núñez-Delgado, A.  (Ref 1) 

 Coccia M. 2010.  (Ref 2)

 Robinson, D. K.. (Ref 3)

 Wang, J., Wu, F., Zhong, H.,Niu, L., Wang, X. 2024.  (Ref 4) 

 Coccia Mario. 2025. (Ref 5)

 Hacker, A. Engel, and M. Mauer, “Regulating ChatGPT and other large generative AI models,” in Proc. ACM Conf. Fairness, Accountability, Transparency, 2023, pp. 1112–1123, doi: 10.1145/3593013.3594067.

Is the argument persuasive and supported by appropriate evidence?

Partly

Is the work clearly and cogently presented?

Partly

Is the topic of the essay discussed accurately in the context of the current literature?

Partly

Does the essay contribute to the cultural, historical, social understanding of the field?

Partly

Reviewer Expertise:

Innovation, science and research policies.

I confirm that I have read this submission and believe that I have an appropriate level of expertise to confirm that it is of an acceptable scientific standard, however I have significant reservations, as outlined above.

## References

[1] Planet Earth: Scientific Proposals to Solve Urgent Issues.2024; 10.1007/978-3-031-53208-5 10.1007/978-3-031-53208-5

[2] : Public and private R&D investments as complementary inputs for productivity growth. *International Journal of Technology, Policy and Management* .2010;10(1/2) : 10.1504/IJTPM.2010.032855 10.1504/IJTPM.2010.032855

[3] : The evolution of mission-oriented policies: Exploring changing market creating policies in the US and European space sector. *Research Policy* .2019;48(4) : 10.1016/j.respol.2018.10.005936-948 10.1016/j.respol.2018.10.005

[4] : Accelerating the establishment of a new science-policy panel to address the triple planetary crisis. *Environmental Science and Ecotechnology* .2024;22: 10.1016/j.ese.2024.100498 10.1016/j.ese.2024.100498 PMC1156553139554833

[5] : Destructive Creation of New Invasive Technologies: Generative Artificial Intelligence Behaviour. *Technologies* .2025;13(7) : 10.3390/technologies13070261 10.3390/technologies13070261

# Reviewer report: Reviewer response for version 1

**DOI:** 10.21956/openreseurope.22576.r59516

**Published:** 2025-09-16

**Authors:** Mar Rubio-Varas

**Affiliations:** 1 Public University of Navarra, Pamplona, Spain

This article provides a valuable historical critique of the Frascati Manual and its role in shaping science policy since the 1960s. The authors make a compelling case that this foundational accounting system has become increasingly inadequate for measuring contemporary research practices, particularly in light of digital technologies, multidisciplinary approaches, and knowledge co-creation.

 The paper offers a well-documented historical analysis of how the OECD's standardized R&D measurement framework emerged and became institutionalized globally. The authors effectively demonstrate how the Manual's original parameters, designed for 1960s research contexts, create systematic biases against social sciences, humanities, and non-traditional forms of innovation. The critique of the Manual's limitations in capturing citizen science, open-source collaboration, and mission-oriented research is particularly relevant for current policy discussions.

 Two significant contextual elements deserve greater attention:

 1.  The development of R&D accounting **should be situated within the broader evolution of national accounting systems** during this period (most notably the whole of the national accounting system, but also other comparable statistics such as foreign investment and the like, in which the OECD played a major role). The Frascati Manual emerged alongside other standardized economic measurement frameworks, and this parallel development would help readers understand why certain methodological choices were made and how they reflected broader trends in economic quantification.

 2. The article would benefit from m **ore substantial discussion of the European Community's own science policy** development, particularly Euratom's nuclear research focus. The EC's research policy, heavily centered on nuclear development through the Joint Nuclear Research Centre (established under the Euratom Treaty), created its own systematic biases against social sciences and shaped European approaches to R&D measurement. This parallel institutional development influenced how European countries implemented OECD guidelines and created additional layers of bias in research accounting.

 The nuclear focus of early European research policy (through programs like ORGEL and the establishment of research centers at Ispra, Petten, Geel, and Karlsruhe) represented a significant alternative model to both UNESCO's internationalist approach and the OECD's economic growth framework. This "third way" merits discussion as it helps explain variations in how different countries adopted and adapted the Frascati framework.

 The paper could benefit from clearer discussion of potential alternatives or reform pathways, given the authors' acknowledgment of the difficulties in changing embedded systems.

 These additions would strengthen the historical analysis and provide a more complete picture of the institutional landscape that shaped R&D measurement systems.

Is the argument persuasive and supported by appropriate evidence?

Partly

Is the work clearly and cogently presented?

Yes

Is the topic of the essay discussed accurately in the context of the current literature?

Yes

Does the essay contribute to the cultural, historical, social understanding of the field?

Yes

Reviewer Expertise:

Economic History, Socio-technical systems

I confirm that I have read this submission and believe that I have an appropriate level of expertise to confirm that it is of an acceptable scientific standard, however I have significant reservations, as outlined above.
